# Retinal crystalline deposits in a patient who received chemotherapy and radiotherapy for nasopharyngeal carcinoma and subsequent anti-VEGF treatment for the bilateral radiation maculopathy

**DOI:** 10.3205/oc000090

**Published:** 2019-02-06

**Authors:** Melih Parlak, Burcin Erden, Ali Osman Saatci

**Affiliations:** 1 Ulm University Medical School, Department of Ophthalmology, Ulm, Germany; 2 Dokuz Eylul University Medical School, Ophthalmology Department, Izmir, Turkey

**Keywords:** cisplatin, crystal, epirubicin, neurodegeneration, radiation maculopathy

## Abstract

We report the occurrence of intraretinal crystalline deposits in a patient who received several anti-VEGF injections and one session of focal laser treatment for the treatment of radiation retinopathy during the treatment process. She had received three cycles of epirubicin and cisplatin together with radiation therapy seven years prior to detection of the maculopathy. The multimodal imaging features and the possible cause of the retinal crystalline deposits are discussed.

## Introduction

Radiation maculopathy (RM) is a sight-threatening consequence of radiotherapy in the management of ocular malignancies or other head tumors. It develops as a slowly progressive retinal microangiopathy and it is histopathologically characterized by vascular remodelling, chorioretinal inflammation, and neurodegeneration [1], [2], [3]. Main clinical features are retinal hemorrhages, microaneurysms, telangiectasia, macular edema, and exudation. The incidence depends mainly on the radiation dose as well as the tumor location and radiation sensitizers [4], [5]. We report an unusual RM case with retinal crystalline deposits during the long-term follow-up of 11 years. 

## Case description

A 27-year-old female patient presented with a progressive visual impairment in both eyes in 2006. She had received charged particle radiation in 1998 for nasopharyngeal carcinoma together with 3 cycles of chemotherapy (each epirubicin 150 mg + cisplatin 150 mg). There was no history of diabetes mellitus, arterial hypertension or any other systemic vascular diseases. 

**Figure 1:**
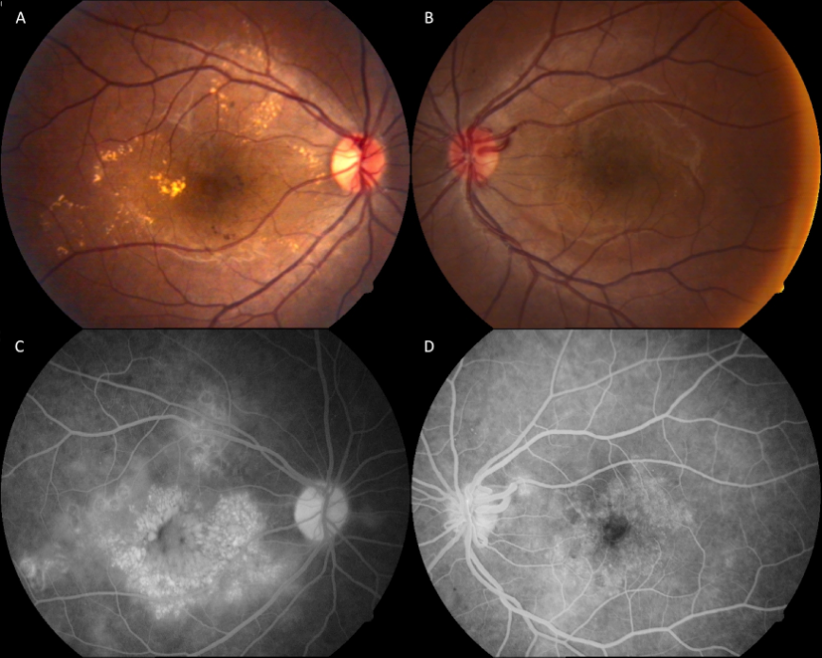
Color fundus photography (initial manifestation): radiation maculopathy with enlarged arterial calibration, intraretinal hemorrhages, exudates, microaneurisms, and diffuse macular edema (A, B) Fundus fluorescein angiography (late venous phase): bilateral enlarged and clear-cut foveal avascular zone with perifoveal teleangiectasia and diffuse macular edema (C, D)

At the initial presentation, the best-corrected visual acuity was 20/100 in the right eye and 20/50 in the left eye (bilateral –3.00 spherical equivalent). The intraocular pressure and slit lamp examination of the anterior segment was unremarkable. Funduscopic examination of the right eye revealed intraretinal hemorrhages, exudates, and a diffuse macular edema. The left retina showed modest intraretinal hemorrhages and microaneurisms at the posterior pole and enlarged arterial calibration (Figure 1 A, B (Fig. 1)). Fundus fluorescein angiography (FFA) revealed an enlarged and clear-cut foveal avascular zone with perifoveal telangiectasia, which was more pronounced in the right eye. Diffuse macular leakage was observed in the late venous phase bilaterally (Figure 1 C, D (Fig. 1)). 

**Figure 2:**
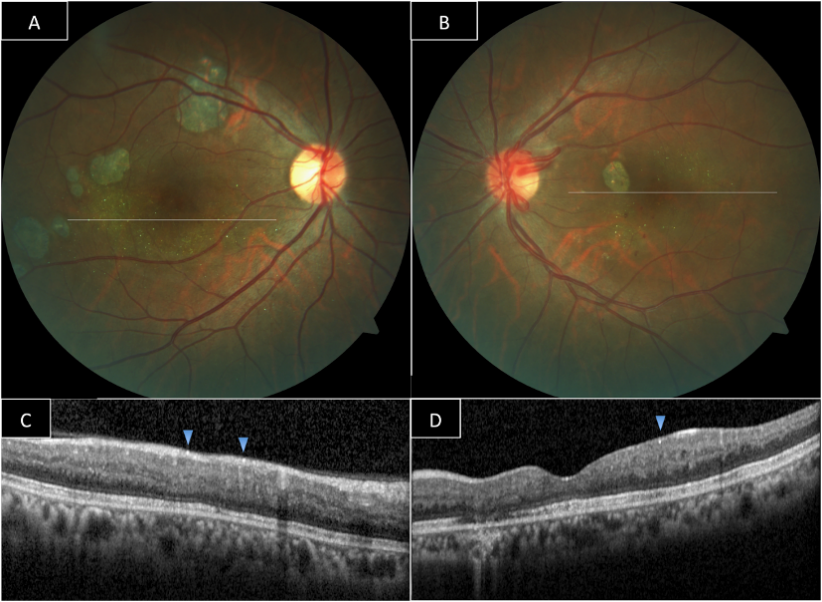
Color fundus photography (Eleven years after initial presentation): retinal crystals surrounding the fovea bilaterally (A, B). Optical coherence tomography (single horizontal linear scan): punctate hyperreflectivity in near all retinal layers. Retinal crystals are corresponding to superficial hyperreflective spots (C, D).

The ischemic macular changes and medical history of radiation therapy suggested us the diagnosis of radiation maculopathy. The patient was subsequently treated with one session focal laser photocoagulation OU and multiple intravitreal injections in between 2006 and 2014 (5 Bevacizumab, 1 Pegaptanib and 6 Ranibizumab injections for OD and 1 Bevacizumab, 2 Pegaptanib, and 2 Ranibizumab injections for OS). Consequently, the macular edema resolved, the visual acuity improved to 20/40 right and 20/30 left, and the patient was followed in longer periodic intervals. During a routine examination in 2013, we noticed retinal crystals, which surrounded and spared the fovea (Figure 2 A, B (Fig. 2)). Optical coherence tomography (OCT) showed hyperreflective dots mainly located in the superficial nerve fibre layer, which corresponded to the crystalline deposits and additionally punctate hyperreflectivity in the outer plexiform and nuclear layer (Figure 2 C, D (Fig. 2)). However, as the old fundus camera was replaced in 2013 all color pictures could not be reviewed. Only the first colour picture captured at the presentation had copies. 

In between 2014 and 2017, functional and morphological findings were stable without necessitating further treatment. However, the crystalline deposits changed throughout slightly. 

## Discussion

Radiation-induced vision loss remains an important challenge in the treatment of ocular oncology. Especially in patients with choroidal melanoma receiving scleral plaque radiotherapy, RM can occur in up to 46% [4]. Therapeutic apical radiation doses in excess of tissue tolerance induce a progressive change in capillary architecture, which contribute to the development and the maintenance of macular edema [1], [2], [5], [6]. Continuous and periodic VEGF inhibitors or intravitreal steroids can be used for preventing vision loss and scarring [7]. 

The clinical course of our case corresponds to a typical RM. The patient noticed visual deterioration seven years after the head radiation. During the follow-up, we observed frequent relapses of macular edema. Common intravitreal anti-VEGF injections were needed to achieve a functionally and morphologically stable retina.

**Figure 3:**
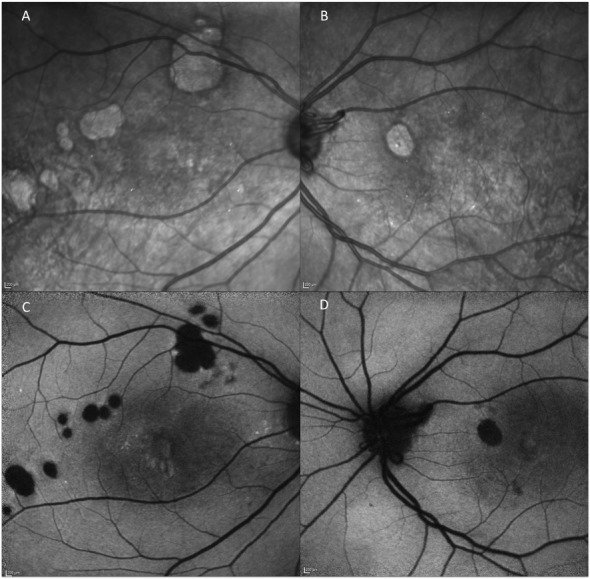
Infrared scanning laser ophthalmoscopy images: punctate hyperreflectivity (A, B). Fundus autofluorescense: hypoautofluorescence due to old laser scars (C, D).

The current case distinguishes itself by retinal tiny crystalline deposits in both eyes, which were observed 7 years after the initial presentation. Interestingly, these crystals showed no autofluorescence and were hyperreflective in infrared scanning laser ophthalmoscopy (SLO) images (Figure 3 (Fig. 3)). Optical coherence tomography revealed a corresponding hyperreflectivity in superficial layers. In the further clinical course these crystals have almost not changed in amount and morphology.

Several clinical entities can contribute to the presence of crystalline deposits in the retina [8]. In our case, we found no laboratory evidence for metabolic disorders or electrolyte imbalances. Specific drug ingestion, like tamoxifen, canthaxanthine, ritonavir or nitrofurantoin, was denied. 

Our patient was previously treated with cisplatin, which has well-known adverse effects such as nephro- and neurotoxicity, especially with intraarterial application [9], [10]. In a recent case-controlled study by Dulz et al. [11], OCT and ERG parameters were analysed in 28 eyes of 14 patients, who were treated with cisplatin. They found a significant correlation between the cumulative cisplatin dose and retinal nerve fiber layer (RNFL) thinning. No significant differences were observed in visual acuity, colour vision or ERG amplitudes, in comparison to controls. Morphological retinal changes like crystalline deposits were not reported in this work.

Retinal crystals can be seen in macular teleangiectasia (MacTel), too, which is an acquired bilateral macular disorder, with a primarily neurodegenerative pathogenesis. It is characterized by minimal dilatation of the parafoveal capillaries, graying of the central maculae and also crystalline deposits in superficial layers. Sallo et al. [12], analyzed the data derived from the MacTel study and reported crystalline deposits in 46% (203 out of 443) of patients. In a multicentre study by Baumüller et al. [13], they analysed the SD-OCT features of 41 patients with mactel type 2. In all eyes SD-OCT revealed distinct hyperreflective spots (HRS) in all retinal layers including the outer retinal layers. Crystalline deposits visualized on fundus photographs, correlated to highly reflective spots in the superficial retina. The authors suggest that these HRS and bands are the results of a nonspecific neurodegenerative process. 

Especially in RM, Frizziero et al. [14] analysed the presence of HRS with SD-OCT. They found them preferentially in the outer plexiform and outer nuclear layer, in all of 25 affected eyes with RM. These HRSs significantly increased in number, according to OCT central subfield thickness. The authors considered HRSs as a biomarker for RM activity, and assessed them mainly as a sign of retinal inflammation. Funduscopic images were not correlated and specific crystals were not reported. 

The precise nature and molecular constituents of the retinal crystals in our case will remain unclear. Various possibilities can be considered, but in view of the morphological similarities with macular telangiectasia we want to suggest a possible neurodegenerative process in the pathogenesis of radiation maculopathy. 

## Notes

### Competing interests

The authors declare that they have no competing interests.
